# Advantage of the go/no-go task over the yes/no lexical decision task: ERP indexes of parameters in the diffusion model

**DOI:** 10.1371/journal.pone.0218451

**Published:** 2019-07-01

**Authors:** Hyeonjin Lee, Yoonhyoung Lee, Jini Tae, Youan Kwon

**Affiliations:** 1 Department of Early Childhood Education, Yeungnam University, Gyeongsan-si, Rep. of Korea; 2 Department of Psychology, Yeungnam University, Gyeongsan-si, Rep. of Korea; 3 Multi-lingualism and Multi-culturalism Research Center, Konkuk University, Seoul, Rep. of Korea; 4 Department of Psychology, Daegu Catholic University, Gyeongsan-si, Rep. of Korea; Universita degli Studi di Roma La Sapienza, ITALY

## Abstract

Previous research findings supporting the advantages of the go/no-go choice over the yes/no choice in lexical decision task (LDT) have suggested that the go/no-go choice might require less cognitive resources in the non-decisional processes. This study aims to test such an idea using the event-related potential method. In this study, the tasks (yes/no LDT and go/no-go LDT) and word frequency (high and low) were manipulated, and the difference between the go/no-go choice and yes/no choice were examined with BP, pN, pN1, P200, N400, and P3 components that were assumed to be closely related with the various parameters in the diffusion model. The results showed that BP, pN and pN1 amplitudes reflecting the preparation stage were not differently affected by word frequency and the task type. However, ERPs after stimulus onset showed differences. The P200 amplitudes were smaller in the go/no-go task than in the yes/no task only for low-frequency words. N400 and P3 amplitudes were only affected by word frequency. The results suggest that the go/no-go task and the yes/no task differ in sub-lexical processes, which is indicated in the *T*_*encoding*_ parameter in the diffusion model. This study is important as it offers the first electrophysiological evidence supporting the assumption in the diffusion model that explains the advantage of the go/no-go choice over the yes/no choice.

## Introduction

A large number of studies have used lexical decision tasks (LDT) based on yes/no choice (or two choices) in order to identify the specific cognitive processes underlying language processes [1, 2]. However, some researchers have noted that the yes/no choice may generate extra errors and biases irrespective of experimental conditions [3]. Although not as popular, go/no-go LDT has been also used to examine cognitive processes during word recognition because the simpler response of the go/no-go LDT generates reduced variability in reaction time (RT), faster RT, and lower error rates compared to the yes/no LDT [4, 5].

In the go/no-go LDT, participants are asked to press the “go” button when a presented string is categorized as a word. Unlike the yes/no LDT, they do not need to press any button if a presented string is categorized as a non-word. While the yes/no LDT asks participants to select “yes” or “no” response obligatorily, the go/no-go LDT requires only one response after deciding whether a presented item is a word or non-word. Therefore, researchers have suggested that the difference in cognitive processes between the yes/no and go/no-go LDT mainly stems from the response selection stage of the task processing than the lexical selection stage where an appropriate item is selected among activated candidates [4, 6, 7]. In the yes/no LDT, participants must remember two response keys matching “yes” and “no” and they could make errors in cases when they forget this mapping or simply press wrong button.

Until lately, few efforts have been made to understand why the go/no-go choice generates lower variability, faster RT, and lower error rate. However, several research have attempted to explain the underlying reasons of the advantage of the go/no-go LDT over yes/no LDT. For example, Allen, Madden, Weber, and Groth [7] suggested that response selection demand of yes/no LDT is responsible for the disadvantage of the yes/no LDT. When they examined age differences in word recognition with yes/no LDT and go/no-go LDT, they found that age differences were larger in yes/no LDT. Navarro-Pardo, Navarro-Pardos, Gamermann, and Moret-Tatay [8] also employed both of yes/no LDT and go/no-go LDT to test the influence of age on visual word recognition. In this study, older group showed longer reaction times and more variability in their responses. However, regardless of age, reaction times and error rates were much lower in go/no-go LDT than in yes/no LDT. They suggested that word frequency rather than the type of task generate the attentional demand. They suggested that go/no-go LDT requires less processing demand but word frequency rather than the type of task generates the attentional cost.

Recently, there have been attempts to examine the advantage of the go/no-go LDT over yes/no LDT based on the diffusion model [9–12]. The diffusion model is a mathematical model that explains decision processes based on reaction times and error rates. This model has successfully explained processing mechanisms of various task paradigms such as memory task, same/different matching task, numerosity judgments, visual-scanning, brightness discrimination, color discrimination, and letter discrimination [13–18]. In this model, it is assumed that the decision about whether a stimulus is a word or a non-word is made by a process that accumulates noisy information over time from a starting point toward one of two response criteria, where the two response criteria are called as *a* parameter and 0, and the starting point is called as the *z* parameter [19]. Total reaction times and error rates are determined by several parameters reflecting underlying cognitive processes, such as the drift rate parameter and the *T*_*er*_ parameter. The mean time for making the decision about whether a stimulus is classified as a word or a non-word is mainly determined by the drift rate, which is a function of the quality of the information extracted from the stimulus.

Since drift rate is defined as the ease of extracting information from a stimulus, the quality of stimulus is critical for deciding drift rates. In case of low frequency words and pseudo-words, it is assumed that it is relatively difficult to extract information, and the absolute value of drift rate is relatively low. On the contrary, the absolute value of the drift rate is relatively high for high frequency words and non-words with illegal strings that are assumed to be relatively easy to be classified. The *T*_*er*_ parameter covers processes that are important yet irrelevant to the decision processes such as encoding (*T*_*encoding*_) and response execution (*T*_*response*_) [19, 20]. Therefore, the overall RT for the decision about whether a stimulus is a word or a non-word is decided by the summation of the decision time (e.g., drift rate) and the non-decision time (e.g. *T*_*er*_).

Studies comparing these parameters of the go/no-go LDT and yes/no LDT have revealed that the difference between the two tasks stems from the *a* parameter and the *T*_*er*_ parameter but that there is no difference in the drift rate parameter for the two tasks [8–12]. For example, Moret-Tatay and Perea [13] performed the go/no-go and yes/no LDT for high and low frequency words with second- and fourth-grade children. While word frequency effect was similar in both the go/no-go and yes/no LDT, children from both grades showed faster RTs and lower error rates in the go/no-go LDT than the yes/no LDT. Based on the absence of any difference in the word frequency effect, Moret-Tatay and Perea [11] concluded that the go/no-go choice had an advantage over the yes/no choice mainly because of the decision criteria setting, and response execution. In addition, Perea, Devis, Marcet, and Gomez [12] compared RTs and the error rates of older and young adults who performed LDT and non-verbal discrimination task with go/no-go and yes/no choices. Although the benefit of the go/no-go choice over the yes/no choice was not found in the non-verbal discrimination task, the go/no-go choice generated faster RTs and lower error rates than the yes/no choice in the LDT. These results can be interpreted to mean that the advantage of the go/no-go choice over the yes/no choice is limited to LDT. However, these results clearly indicate that the advantage of the go/no-go choice over the yes/no choice was not induced from the rate of the information extraction.

As explained above, several behavioral studies have examined the benefit of the go/no-go choice over the yes/no choice. However, to the extent of our knowledge, no study using event-related potential (ERP) has been conducted with regard to this topic. This is somewhat surprising, since the diffusion model’s assumption of the stages of processes is related to time lapse (see Fig 1 in [19]). Since reaction time results in behavioral research can only reflect the total processing time from encoding of information to motor execution for a response, behavioral studies are limited in terms of identifying a specific underlying cognitive process.

Because, the ERP method has a very good temporal resolution to provide more delicate evidence about the underlying cognitive function [21], we believe that the ERP method could provide more information to help identify the source of the advantage of the go/no-go choice over the yes/no choice and the underlying cognitive processes associated with the parameters in the diffusion model. Therefore, the present aimed to examine the underlying reasons of the advantage of the go/no-go LDT over the yes/no LDT using the ERP method. As far as we know this is the first ERP study examining whether a specific parameter of the diffusion model can be reflected in a specific ERP component.

To find the neurological differences and the underline cognitive functions between two tasks, we focused on several ERP components that have been generated in different-time windows. We tested ERP components reflecting motor and cognitive preparation (BP and pN), visual encoding (pN1), language processing (P200, N400), and task demand (P3).

To perform the go/no-go LDT or the yes/no LDT, the participants have to cognitively and behaviorally prepare to respond to each trials. Such preparation can be shown in specific ERPs before the actual responses. Therefore, the first ERP that we were interested in was Bereitschaftspotential (BP) or readiness potential known to be related with motor preparation [22,23]. BP is usually shown as a gradient negativity around 500 ms before movement onset at prefrontal electrode sites (e.g., Fp1 and Fp2).

In the same time window, another ERP component which is called as prefrontal negativity (pN) can be observed. It is known as index of the cognitive preparation of the response [22]. While the two ERP components can be identified in the same time window, the brain sources are different. The brain source of BP is somatosensory cortex and pN comes from the bilateral inferior frontal gyrus [24]. Since the BP and pN components are indexes of the motor and cognitive preparation, we assume that they are closely matched onto the starting point parameter (z) of the diffusion model. If there is any different at the preparation stage between the go/no-go LDT and yes/no LDT, we would see different in modulation of the two components of the two tasks.

The next ERP component that we focused on was the prefrontal N1 around 200 ms (pN1). This ERP component is generated after target onset and is tended to be modulated by factors affecting visual encoding such as luminance, contrast, and spatial attention [25]. The stimulus presentation method was the same in the go/no-go LDT and yes/no LDT. Therefore, we expected little difference in the visual encoding processes and the modulation of the pN1 of the two tasks. However, since recent study suggested that pN1 can be differently affected by the type of task [20] and *T*_*encoding*_ parameter in the diffusion model might reflect a general visual encoding process, we decided to take a close look at the component as well.

The earliest ERP component reflecting linguistic aspect of the LDT process was the P200 component that was to be related with the lower-level linguistic processes [26–28]. This component is the positive going peak of the waveform within the 150 ms and 300 ms time window. In psycholinguistic studies, P200 has been considered as the ERP component to be modulated by the characteristics of syllable units. Barber, Vergara, and Carreiras [26] found that the size of the P200 amplitude was negatively related with the syllable frequency of the first syllable in a multisyllabic word. Other study such as Kwon, Lee, and Nam [28] found that the modulation of P200 component was sensitive to phonological features when mapping orthographic representation onto phonological representation. In addition, Kwon, Nam, and Lee [29] observed that P200 is independent of the morphological processing that is involved in higher-level processing. In attention studies, P200 is a well-known to be associated with early attention-related processes before categorization processes [30, 31]. Since Näätänen [32] demonstrated that the P200 component might be related to the processes of allocating attention, considerable studies have shown that increase in the level of attentiveness generates a decrease in the P200 amplitude [31].

Considering previous studies from psycholinguistic studies and attention studies, we believe that P200 is an index of ERP component for attention allocation, lower level sub-lexical encoding and syllable parsing. Therefore, if the difference between the go/no-go LDT and yes/no LDT can be reflected in the *T*_*encoding*_ parameter that covers encoding processes, we expect that the difference between the two tasks will be shown in the different modulations of the P200 components of the two tasks.

The second linguistic ERP component of interest to us is the N400 component that shows a negative going peak around 400 ms after target onset. It is well-known to be sensitive to lexical features such as word frequency and other semantic features of presented items [33, 34]. Given that the relationship between N400 and word frequency is robust and that the drift rates parameter in the diffusion model are very closely related with the word frequency effect [35, 36], we assumed that N400 would reflect the drift rates parameter. Additionally, since the diffusion model expects no difference in the size of the word frequency effect between the yes/no LDT and go/no-go LDT, we expected that the word frequency effect measured using the difference in the size of N400 component in low frequency words and high frequency words would be very similar in the two tasks. To test this idea, the present study manipulated two factors, namely, word frequency (high and low) and the task (yes/no LDT and go/no-go LDT).

The last ERP component is P3, which is elicited 250 to 1000 ms after stimuli onset. It is known that frequent targets generate larger P3 amplitude than infrequent targets in oddball paradigm. Therefore, the P3 is often described as a component that is elicited by uncertain or surprising stimuli [37]. However, the size of P3 amplitude is also known to be related with response types [38]. Even when the two stimulus categories occur in the same frequency, larger P3 was generated when participants were required to provide go-responses in the go/no-go task [39]. A similar pattern was observed in response inhibition trials [40] and a silent counting task [41]. Moreover, P3 latency is sensitive to the reaction time of how rapidly participants respond to the targets. Kida et al. [42] found that P3 latency was positively related with RTs when they asked participants to response for targets as rapidly as they could. Based on such findings, the present study assumed that the difference between the go/no-go LDT and yes/no LDT task would be shown in the P3 component, if the difference between two tasks mainly originates from the processing of the response execution. However, if the advantage of the go/no-go choice over the yes/no choice is not based on the response execution difference, there would be no difference of the P3 modulation between the two tasks. This finding suggests that the response execution part of the *T*_*response*_ parameter in the diffusion model does not reflect the difference between the two tasks.

## Method

### Participants

This study was approved by the Institutional Review Board (IRB) of Konkuk University(7001355-201403-HR-006). A total of 24 students (7 females) were recruited from the Konkuk University. Participants were recruited through bulletin boards and as results, there were more male participants. We assumed that it is because participants were informed that they needed to wash their hair after the experiment. Although it would not be a critical, the discrepancy between number of male participants and female participants could cause possible bias even in minor degree.

They were right-handed and native speakers of Korean. Their ages ranged between 19 and 28, and they had normal or corrected-to-normal vision. They reported that they had no history of neurological diseases. All participants signed a written consent form before participating the experiment. They were paid 15,000 won (about $13) for their participation.

### Stimuli

A total of 120 bisyllabic words (60 high and 60 low frequency) were selected from the Korean Word Database [43]. Across conditions, the number of orthographic neighbors, bigram frequencies, the number of more frequent orthographic neighbors, and the syllable frequencies of target words were controlled (see Table 1). A total of 100 non-experimental words from the same database were included to prevent participants from forming any strategy. The range of frequency of fillers was 6 to 553. To conduct lexical decision tasks, 220 non-words were created. A total of 110 non-words were created by replacing one letter from legal words, and another 110 non-words were created using illegal strings based on the Korean orthographic rule.

**Table 1:** Features of words.

Features	High frequency		Low frequency	
	Mean	SD	Mean	SD
Word Frequency	222.7	74	13.9	3.1
Number of more frequent orthographic neighbors	0.2	0.1	0.2	0.1
Number of orthographic neighbors	0.4	0.1	0.4	0.7
Bigram frequency (log)	2.5	1.7	2.5	1.8
1st syllable frequency (log)	2.1	2	2.2	2
2nd syllable frequency (log)	2.0	1.9	1.9	1.7
Number of letters	5.3	0.5	5.2	0.4

### Procedure

Before the experiment, written informed consent was obtained from all participants. Each participant performed both the go/no-go LDT and the yes/no LDT. Half of the participants performed the yes/no LDT first and the remaining performed the go/no-go LDT. Between the two tasks, participants took a short break. In both tasks, each trial began with a fixation mark (+) that appeared on the center of the screen for 500 ms. The mark was then replaced with a target string. If the target string was a legitimate Korean word, participants were asked to press the “yes” button in the yes/no LDT and the “go” button in the go/no-go LDT as quickly and accurately as possible. However, if the target string was not a legitimate Korean word, they were asked to press the “no” button in the yes/no LDT as quickly and accurately as possible and to not press any button in the go/no-go LDT. In case of the go/no-go LDT, the target disappeared after 1500 ms unless the participants pressed the “go” button. As commonly used in ERP studies, a 2000 ms interval was presented between trials to prevent noises from the previous trial affecting the ERPs of the current trial [44].

There were 50% of the go trials and 50% of the no-go trials in the go/no-go task. While there are many studies with 50/50 of go/no-go proportion [7, 8, 45–47], some researchers advised that no-go trials should be less than 20% and trial duration should be shorter than 1500ms in the go/no-go task. However, the reason for the recommendation was to accurately examine inhibitory control activity of no-go trials [48]. Since no-go trials were not the main interest of the current study and the main purpose of the current study was to examine the advantages of the go/no-go choice over the yes/no choice in LDT by comparing the “yes” response of the yes/no task and the “go” response of the go/no-go task, we choose to have 50/50 of go/no-go proportion in the go/no-go task just as the yes/no task. If we adapt different proportion of “yes” and “go” trials in the yes/no and the go/no-go task, it would cause response bias toward a certain response.

### EEG (Electroencephalogram) recording

**Fig 1:**
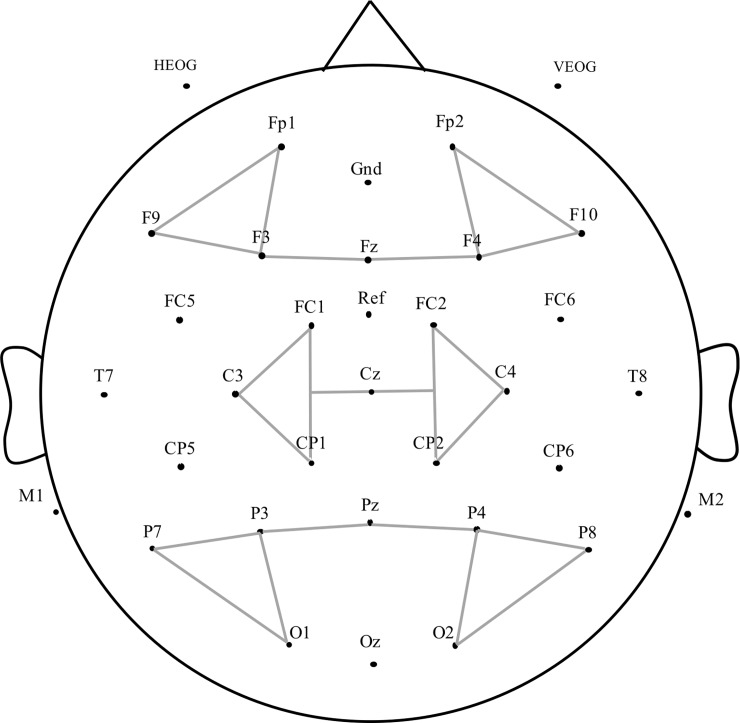
Montage of electrodes.

Scalp voltages were collected using 32 actiCAP electrodes. All the EEGs were amplified by a Brain Products’ Standard Brain Amp with an analog bandpass filter of 0.01–100 Hz. A channel located between Fz and Cz was used as an online reference channel, and two mastoid channels were used for the re-referencing purpose when we computed averaging epochs. The electrode below the right eye and the electrode beside the left eye were used to monitor eye blinks and eye movements. The sampling rate was 500 Hz and the impedance level was kept below 10 kΩ. Fig 1 describes the location and names of electrodes.

### EEG data analysis

The present study conducted two separate analyses; ERPs before target onset and ERPs after target onset. The analyses for ERPs before target onset were attempted to find the effect of BP and pN (motor and cognitive preparation) across conditions. The analyses for ERPs after target onset were attempted to find the effect of pN1, P200, N400, and P3 (from encoding to decision). All ERP analyses were performed after band-pass filter (0.01~30Hz) was applied to continuous EEG.

### ERPs before stimulus onset

To compare ERPs before target onset, we adapted analysis methods in Berchicci, Spinelli, and Di Russo [22]. Based on Berchicci et al. [22], we selected epoch from 1500 ms before target onset to 500 ms after target onset and used the baseline from -1000 ms to -800 ms of continuous EEG. In order to obtain clean averaged epochs, epochs containing eye blinks and movements were excluded from the averaging procedure. In this artifact rejection procedure, less than 3.6% of the total epochs were removed. After artifact rejection, averaged ERPs were computed based on word frequency conditions and tasks. For the BP component, mean amplitudes from -500 ms to 0 ms were calculated at Fp1 and Fp2. For the pN component, mean amplitude from from -500 ms to 0 ms were calculated at Fz. 2 (word frequency: high, low) × 2 (task: go/no-go, yes/no) repeated measure analyses were conducted to examine the modulation differences in the BP and pN components. In addition, pN1 at Fz after target onset was also analyzed for the same purpose. For pN1 component, mean amplitudes from 50 ms to 150 ms were calculated at Fz. Although pN1 component is an ERP component after target onset, previous studies suggested that pN1 effect is more sensitively detected when using the same baseline of BP and pN [22, 23]

### ERPs after stimulus onset

To analyze ERPs after target onset, separated epochs from the 200 msec before the target onset to 800 msec after the target onset were selected. After artifact rejection (< 9%), Separated ERPs were formed based on the word frequency conditions, the participants, and the electrode sites. The 200 msec before the target onset was used as the baseline. For increasing statistical power, the three regions of the electrodes were pooled. The regions were anterior (Fp1, Fp2, F9, F10, F3, F4, Fz), central (FC1, FC2, C3, C4, CP1, CP2, Cz), and posterior (P3, P4, P7, P8, O1, O2, Pz). Since the hemispheric difference was not the main interest, we did not pool electrode sites based on the left and right regions (see Fig 1). Analyses were performed to test the effects of the word frequency, the task, and their interactions based on ERPs of the “go” and “yes” response of the two tasks in three-time windows (150–300 ms for P200, 300–500 ms for N400, and 400–800 ms for P3). Therefore, 2 (Word frequency: high, low) × 2 (Task: go/no-go, yes/no) × 3 (Region: anterior, central, and posterior region) repeated measures of variance (ANOVA) were applied. The mean amplitude for three-time windows was the dependent variable. The peak latency of P3 was also calculated for task effect as well.

## Results

### Behavioral result

RTs below 250 ms or over 1500 ms were removed from the analyses (< .7%), and error responses also were excluded from the analyses. Table 2 describes mean lexical decision latencies and error rates. Analyses of variance (ANOVAs) were conducted on the basis of a 2 (Task: yes/no vs. go/no-go) × 2 (Word Frequency: high vs. low) design. Both task and word frequency were within-subject factors.

**Table 2:** Mean reaction times and error rates in the go/no-go and yes/no LDT.

	Go/no-go		Yes/no	
	RT	Error	RT	Error
High frequency	565(81.0)	2.5 (3.7)	576(67.9)	3.3 (3.6)
Low frequency	626(105.4)	8.7 (6.6)	631(95.3)	9.1 (10.1)

In analyses for lexical decision latencies, the word frequency effect was significant (F (1, 22) = 4.01, η^2^ = .15, p < .05) and the difference between tasks was also significant (F (1, 22) = 4.55, η^2^ = .17, p < .05). However, the interaction of the two factors did not approach significant level (p > .10). The pattern of lexical decision latency in the word frequency and task was similar with the previous results reporting relatively faster reaction time in high frequency words over low frequency words and in the go/no-go choice over the yes/no choice. In analyses for error rates, high frequency words showed lower error rates than did low frequency words (F (1, 22) = 21.508, η^2^ = .181, p < .001). Unlike lexical latencies, the main effect of the task and the interaction approached significant level (all ps > .30).

To test the idea that simpler response of the go/no-go LDT causes less variability [49], we compared standard deviations of the participants’ lexical decision latencies and error rates across conditions. Corresponding with the previous suggestion, the standard deviation variation of lexical decision latencies was smaller in the go/no-go task than in the yes/no task (F (1, 23) = 4.731, η^2^ = .170, p < .05). However, for the error rates, the standard deviation variation differences between the two tasks were not significant. As expected, the standard deviation variation of lexical decision latencies was smaller in the high frequency words for both lexical decision latencies (F (1, 23) = 22.203, p < .001, η^2^ = .491) and the error rates (F (1, 22) = 31.568, η^2^ = .185, p < .001). In both lexical decision latencies and the error rates, the interactions of the two factors were not significant (all ps > .10).

### ERP result

#### BP, pN and pN1 effects

**Fig 2:**
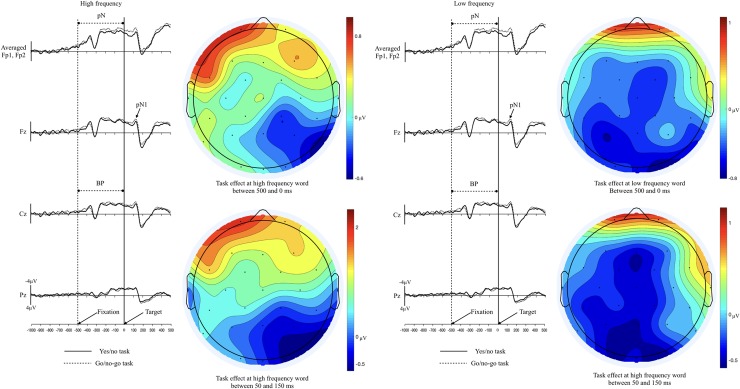
Grand averaged ERPs of the high and low frequency words in the go/no-go task and the yes/no task. Vertical line indicates the target onset and dotted line represents the fixation mark onset. pN(prefrontal negativity); BP(Bereitschaftspotential); pN1(prefrontal N1).

**Fig 3:**
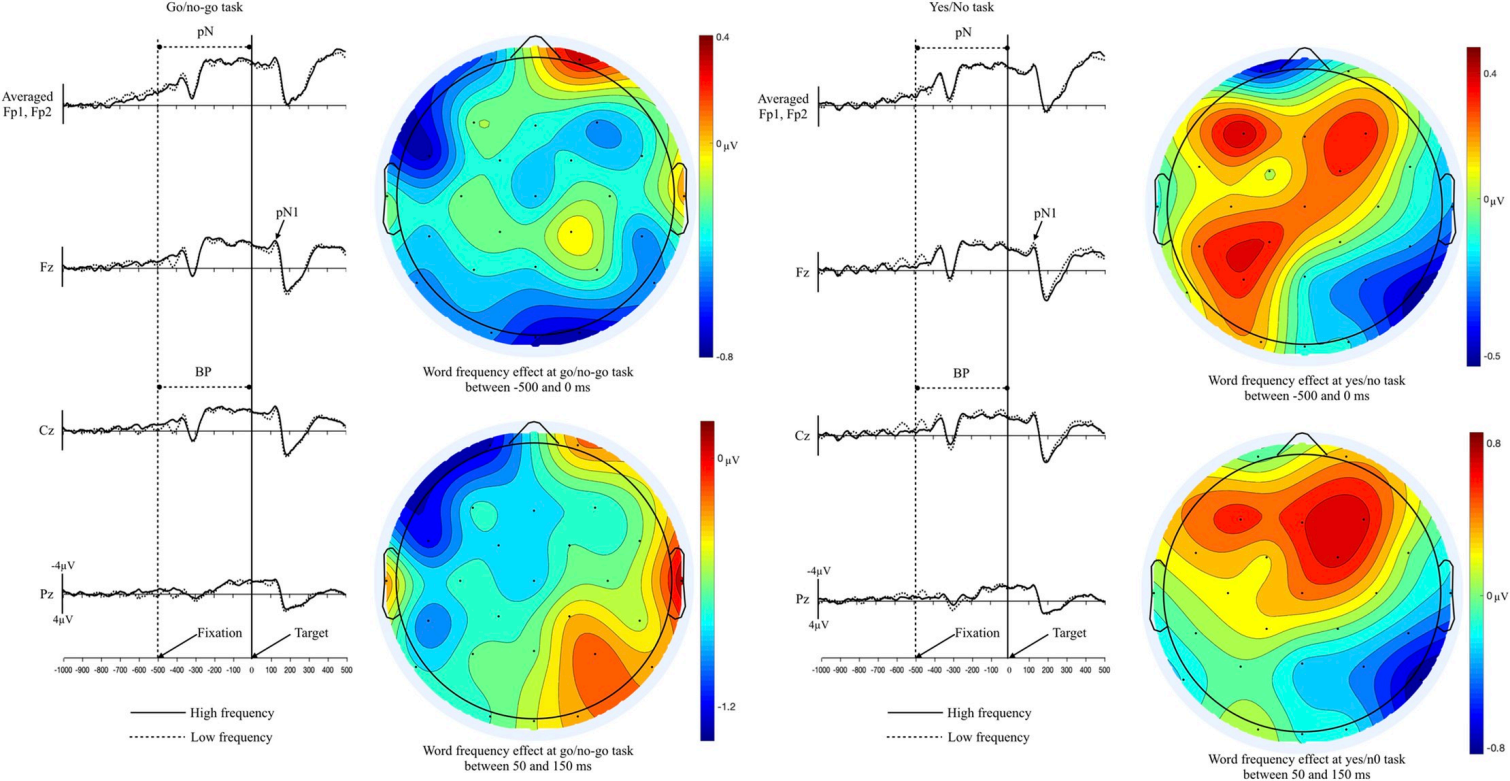
Grand averaged ERPs of the go/no-go task and the yes/no task in the high and low frequency words.

As can be seen in Figs 2 and 3, no significant was found from the BP and pN components (all ps > .1). The peak around 150ms after target onset (pN1) was clearly shown, but the modulation of the component did not differ across experimental conditions (all ps > .1).

#### P200 effect

**Fig 4:**
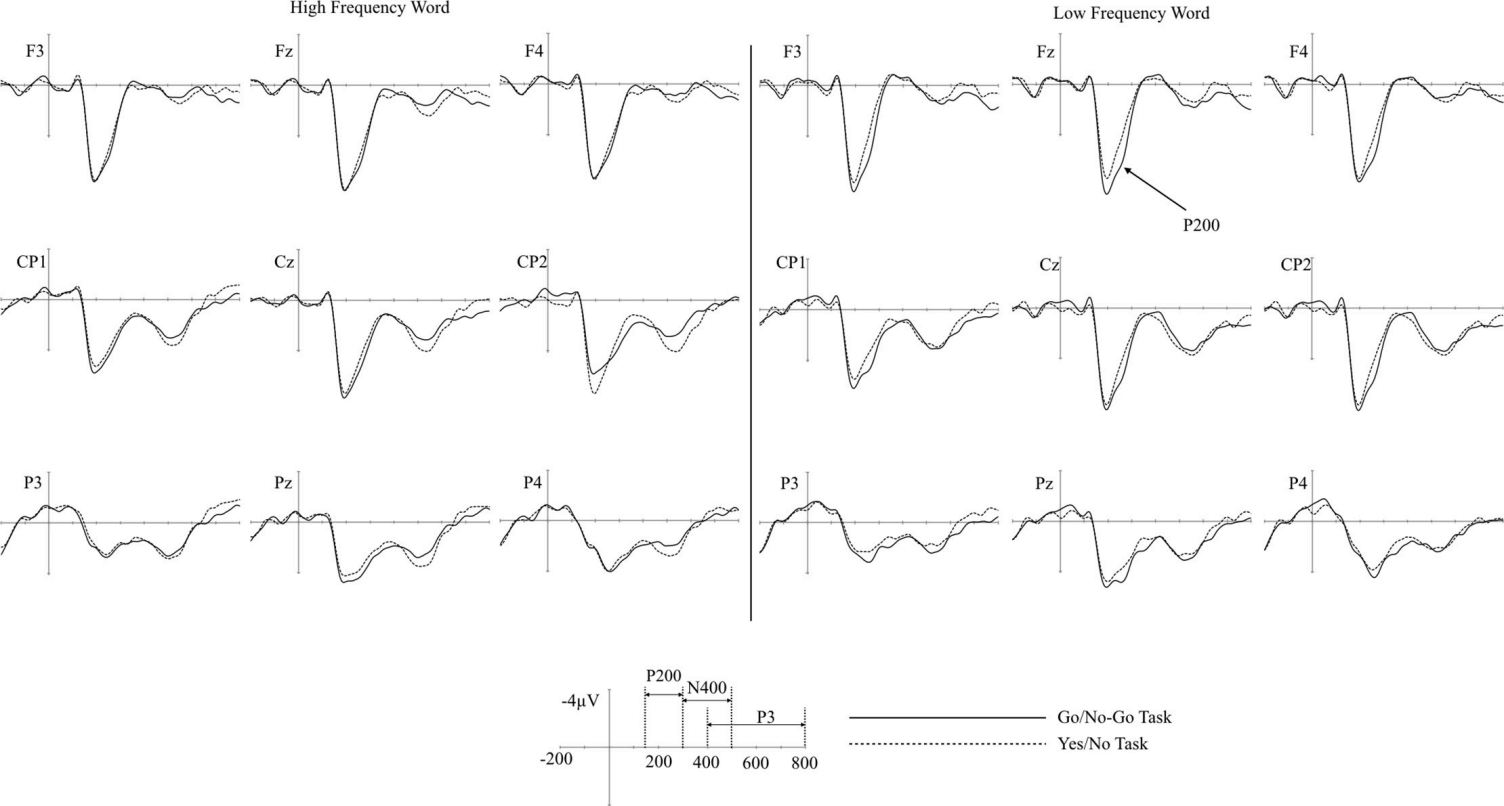
The grand averaged ERP of the high and low frequency words in go/no-go and the yes/no tasks.

**Fig 5:**
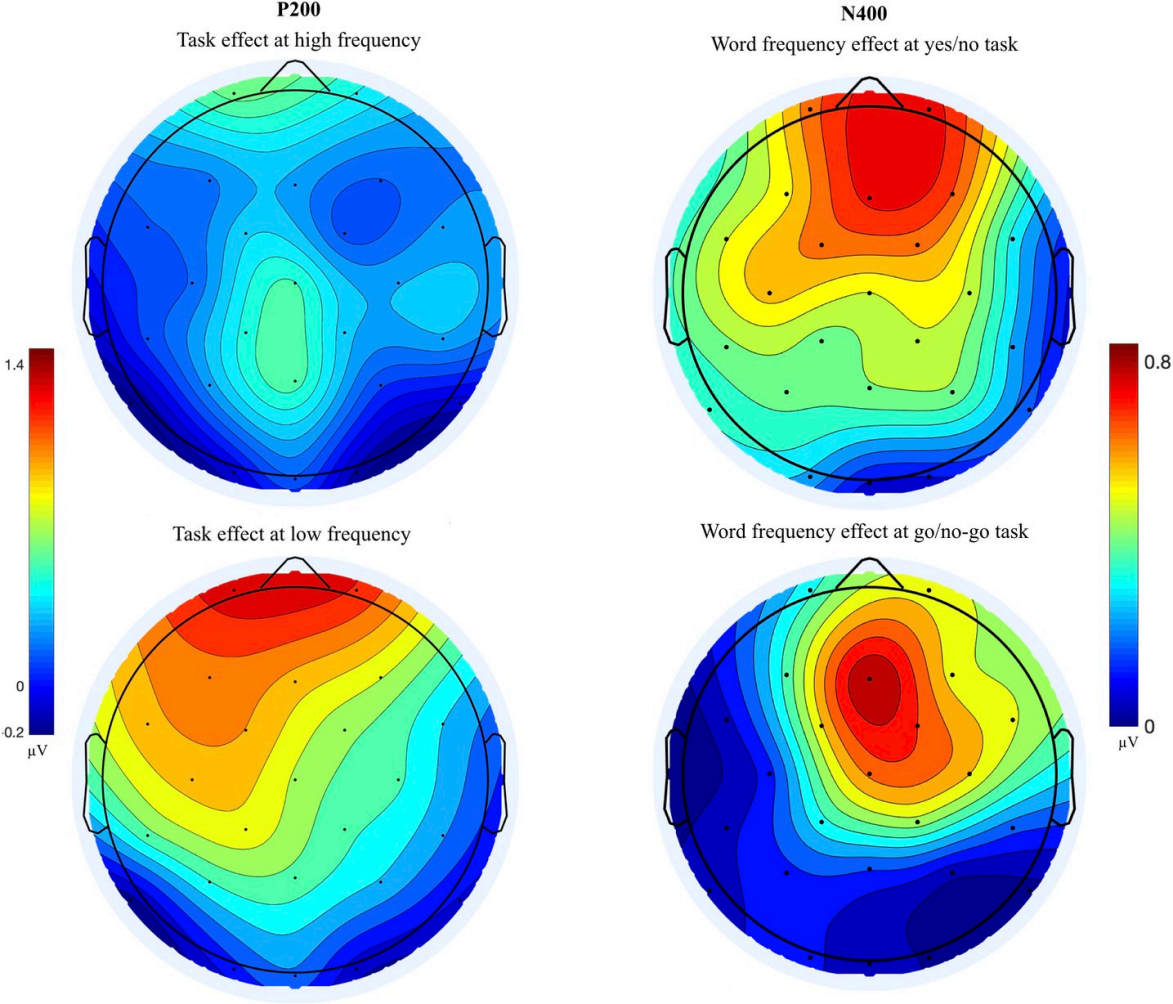
**Left: Topographical map of P200 of the high and low frequency words (subtracting go/no-go from yes/no)**, **Right: Topographical map of N400 of the go/no-go task and the yes/no task (subtracting low frequency words from high frequency words)**.

As can be seen in Fig 4, the P200 difference between two tasks was mainly detected in the low frequency words; the P200 amplitudes were larger in the go/no-go task than in the yes/no task (see. Table 3). From 150 to 300 ms, the main effect of the task was significant (F (1, 23) = 5.121, η^2^ = .0008, p < .05), but the main effect of word frequency was not significant (F (1, 23) = .268, η^2^ = .0002, p = .609). The interaction between the task and the word frequency was significant (F (1, 23) = 4.305, η^2^ = .002, p < .05). In post-hoc analysis for the interaction, the task effect was significant in the low frequency words (p < .01), while other comparisons were not significant (all ps > .1). The main effect of the cluster or other interactions was not significant (all ps > .20). Topographical map of the P200 showed that task effect in the low frequency words was more concentrated in prefrontal sites as compared with that of the high frequency words (Fig 5 left column).

**Table 3:** Mean amplitude (mean peak latency) and standard deviation of all ERP components.

ERP component	Go/no-go		Yes/no	
	High	Low	High	Low
BP(Cz)	-2.39(2.34)	-2.56(1.91)	-2.56(2.45)	-2.13(1.89)
pN(Fp1, Fp2)	-5.50(4.43)	-5.17(6.06)	-5.49(7.58)	-5.6(5.65)
pN1(Fz)	-3.04(4.02)	-3.91(3.61)	-3.77(3.61)	-2.95(3.33)
P200	3.88(3.08)	4.15(3.25)	3.68(3.04)	3.55(2.75)
N400	1.02(1.57)	0.65(1.61)	1.00(1.66)	0.56(1.35)
P3	0.73(1.14)	0.88(1.28)	0.66(1.40)	0.63(1.27)
P3 peak latency	564(115.01)	579(108.46)	565(103.15)	579(102.55)

Since there are no main effect of cluster in P200, N400, P3 and P3 peak, values of P200, N400, P3 and P3 peak latency are averaged on frontal, central and posterior region.

#### N400 effect

**Fig 6:**
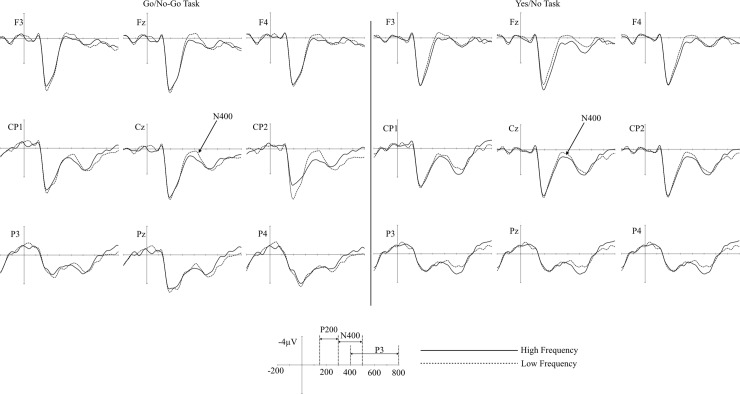
The grand averaged ERP of the go/no-go and the yes/no tasks in high and low frequency words.

As can be seen in the grand average ERPs in Fig 6, larger N400 waveform was shown in low frequency words than in high frequency words in both the go/no-go LDT and the yes/no LDT. Similar to previous studies, N400 is larger in low frequency words than in high frequency words. However, the sizes of the word frequency effects were similar in two tasks. This observation is supported by and statistical results.

As expected, the word frequency effect was significant (F (1, 23) = 5.345, η^2^ = .02, p < .05) but no difference was found in the word frequency effect across the two tasks (F (1, 23) = .136, η^2^ = .0004, p = .71). The main effect of the cluster and all interactions were not significant (all ps > .10). As was expected in the present study, no difference was found in the word frequency effect across two tasks. In topography (Fig 5 right column), the word frequency effect in yes/no task was more widely spread from prefrontal to central electrode sites while the effect in go/no-go task was more concentrated in frontal sites.

#### P3 effect

In the analysis for mean amplitude, no significant effects were found in the word frequency factor (F(1, 23) = .238, η^2^ = .0008, p = .63), task type factor (F(1, 23) = .978, η^2^ = .0004, p = .33), and their interaction (F(1, 23) = 95, η^2^ = .001, p = .339). Since P3 latency was known to be positively related with RT, analysis of the peak latency differences was also performed in this time window. However, the results of the analysis for peak latency were not significant (all ps > .10). Table 3 presented the mean amplitudes of all ERP components across conditions.

## Discussion

The diffusion model has the scalability to apply to human behaviors. Until now, parameters in the diffusion model have been constructed and estimated in terms of RTs and error rates from behavioral tasks. While relatively many studies using the behavioral method have been conducted, no study so far has examined the connection between parameters in the model and the particular cognitive function with ERP method. Therefore, the purpose of current study was to identify the underlying reasons for the advantage of the go/no-go LDT over the yes/no LDT with ERP method from the prospective of the diffusion model.

The diffusion model [3] suggests that the advantage of the go/no-go choice over the yes/no choice originates from encoding and response execution rather than from the information extraction process. Therefore, it assumes that the difference between the tasks should mainly be seen as rapidity of overall responses, lower error rates, and the less variation of data while there should be no significant difference for the size of word frequency effect. This assumption is partially confirmed in our behavioral results. As expected, we found shorter lexical decision latencies and smaller variation in lexical decision latencies in the go/no-go LDT over the yes/no LDT, while there was no difference of the word frequency effect between the two tasks. However, we could not observe reliable benefits in the go/no-go choice for error rates. Perea, Gomez, and Fraga [50] also did not find the benefit of the go/no-go task over the yes/no task, and Hino and Lupker [51] even reported longer lexical decision latency in the go/no-go task over the yes/no task. These results suggest that the difference between the two tasks are relatively small and not easy to observe in behavioral studies.

In the ERP results, the first finding was that there was no difference in BP, pN and pN1. Such results suggest that there is no difference at the preparation stage and visual encoding for performing the go/no-go task and the yes/no task. More important finding was that the size of the P200 amplitude was sensitive to the task type when the presented word had low frequency. The P200 amplitudes are smaller in the go/no-go task than in the yes/no task only for low frequency words while there was no difference for high frequency words. The lower P200 amplitude for low frequency words in the go/no-go task suggests that the go/no-go task facilitates attention allocation and sub-lexical processes such as encoding and syllable parsing.

Corresponding with the results of the current study, previous ERP studies also found that the P200 amplitude is affected by the sub-lexical aspects of a word when the word has relatively low frequency. For example, Carreiras et al. [27] presented colored words and pseudowords and tested if matching the syllable boundaries and the color boundaries had an effect on visual word processing. They found that cases with mismatched syllable boundaries and color boundaries created larger P200 amplitudes for low frequency words and pseudowords. They suggested that syllable parsing processes for low frequency words and pseudowords might be interrupted by the mismatched color-syllable while the interruption might not occur in high frequency words because the syllable parsing for high frequency words could be conducted routinely. In our knowledge, present study is the first to find the ERP difference in the P200 component between the go/no-go and the yes/no task. This result implies that the two tasks lead to different sub-lexical processes or attention allocation and the P200 component can be the ERP index of the *T*_*encoding*_ parameter to reflect the sub-lexical access processing in the diffusion model.

While we found a clear effect of word frequency in the N400 time window, the two tasks showed similar sizes for the N400 difference between the go/no-go task and the yes/no task. The absence of the N400 effect difference between tasks implicates that the semantic information extraction processes between the two tasks were quite similar. Although no supporting electrophysiological results exist yet, our results were in line with previous behavioral studies on LDT that did not find any difference between tasks [7–12, 50]. No interaction between task type and word frequency effect in the present study can be regarded as neurological evidence supporting the hypothesis of the diffusion model that two tasks do not differ in the rate of information extraction.

The difference between the go/no-go LDT and yes/no LDT task was absent in the P3 component as well. There are several reasons for why there was no significant difference between the two tasks in the present study. The first explanation is that, unlike the suggestion in the diffusion model, there is no significant difference in the response execution of “go” in the go/no-go choice and “yes” in the yes/no choice since the participants need to press the button in both cases. Based on this explanation, the current results suggest that the advantage of the go/no-go choice over the yes/no choice is not a consequence of the response execution difference, and the *T*_*response*_ parameter in the diffusion model does not reflect the difference between the two tasks.

Another possibility is that the underlying cognitive function of P3 may not be the response execution. P3 has been mostly observed in the less frequent targets and the unexpected target in a context, such as the oddball paradigm. Additionally, it is well known that P3 is composed of subcomponents, such as P3a and P3b. P3a is called novelty P300 indicating automatic attention orientation for a novel item in the context of unattended stimuli. P3b is related with attended, task-relevant stimuli that are rare and novel [52]. In both tasks in the current study, there were 50% words and 50% non-words. Additionally, it is not necessary to allocate additional attention after the information extraction processes of a given stimuli are already completed. Therefore, neither the P3a nor P3b might be a sensitive ERP component for lexical decision tasks with the stimulus list that has same proportion of words and non-words.

## Conclusion

Previous behavioral studies suggested the advantage of the go/no-go choice over the yes/no choice comes from taxing less cognitive resources in the non-decisional processes. With using more sensitive ERP method, the current study examined the source of the advantage of the go/no-go LDT over yes/no LDT. The results of the current study showed that the advantage of the go/no-go choice over the yes/no choice is not originate from the information extraction processes. The results clearly indicated that the core component of the LDT is not different across tasks. The results also showed that the advantage of the go/no-go choice over the yes/no choice does not come from the response selection processes. Rather, the results suggested that the difference of the task demand between the go/no-go task and the yes/no task mainly comes from the lower level sub-lexical processes and attention allocation that are reflected in the *T*_*encoding*_ parameter in the diffusion model.

As far as we know, this study provides the first electrophysiological evidence supporting the assumption in the diffusion model that explained the advantage of the go/no-go choice over the yes/no choice. This study also the first one presents a close relationship between a specific parameter in the diffusion model and a specific ERP component. We believe that consistent finding of neurological index reflecting the parameters of the diffusion model would validate the parameters and enhance the understanding of the model.

## Supporting information

S1 File Erps_postonset.zip: Erp files of 24 participants. From 200ms before stimulus onset to 800ms after stimulus onset.
(ZIP)

S2 File Erps_preonset.zip: Erp files of 24 participants. From 1500ms before stimulus onset to 500ms after stimulus onset.
(ZIP)
